# The −1154 G/A VEGF gene polymorphism is associated with the incidence of basal cell carcinoma in patients from northern Poland

**DOI:** 10.1007/s00403-014-1471-9

**Published:** 2014-06-06

**Authors:** Michał Sobjanek, Monika Zabłotna, Aleksandra Lesiak, Igor Michajłowski, Aneta Szczerkowska-Dobosz, Małgorzata Sokolowska-Wojdylo, Roman Nowicki

**Affiliations:** 1 Department of Dermatology, Venereology and Allergology, Medical University of Gdańsk, ul. Debinki 7, 80-952 Gdańsk, Poland; 2 Department of Dermatology and Venereology, Medical University of Łódź, Łódź, Poland

**Keywords:** Basal cell carcinoma, VEGF, Vascular endothelial growth factor, Gene, Polymorphism

## Abstract

Vascular endothelial growth factor (VEGF) is believed to play a crucial role in neoplastic angiogenesis. Although the genetic background of basal cell carcinoma (BCC) has been analyzed in some papers, the mechanism of BCC pathogenesis is not fully understood. To the best of our knowledge, *VEGF* gene polymorphisms have not yet been explored. The aim of the study was to asses the frequency of three polymorphisms in the *VEGF* gene (−1154 G/A, −460 T/C and +405 G/C) in patients of Polish origin with BCC and control group. In addition, VEGF serum levels of patients with BCC and controls were measured. The study involved 180 patients (96 women, 84 men) with BCC and a mean age of 68.9 ± 11.8, and 215 healthy age- and sex-matched volunteers. The *VEGF* polymorphisms at positions −1154 and +405 were analyzed using the amplification refractory mutation system polymerase chain reaction method. To assess the *VEGF* gene polymorphism at position −460, we used the polymerase chain reaction restriction fragment length polymorphism method. Serum levels of VEGF protein were measured using the ELISA test. The presence of the G allele (GA or GG) in the −1154 *VEGF* polymorphism was associated with an increased risk of BCC development (OR = 7.28, *p* < 0.0001). Furthermore, the carriers of the AA genotype in −1154 *VEGF* polymorphism showed significantly reduced risks of BCC (OR = 0.14, *p* < 0.0001). It was also shown that the GTC haplotype of *VEGF* predisposes to BCC development (OR = 1.69, *p* = 0.013), while the presence of the ATG haplotype significantly reduces this risk (OR = 0.17, *p* = 0.00001). We have found significantly increased VEGF serum levels among BCC patients, in comparison with the healthy controls (mean 596.7 ± 393.5 pg/ml; range 60.1–931.4 vs. 255.9 ± 174.6 pg/ml; range 42.2–553.0 pg/ml; *p* < 0.0004). The serum levels of VEGF significantly correlated with tumor size: *r* = 0.41, *p* < 0.0001. Our results testify to the importance of −1154 G/A *VEGF* gene polymorphisms in altering the risk of BCC among the population from northern Poland.

## Introduction

Angiogenesis plays the main role in local tumor growth and invasion, as well as in metastasis. It is known that, without the formation of new vessels, tumors cannot exceed 1–2 mm in diameter [2–4].

Vascular endothelial growth factor (VEGF) is believed to play a crucial role in neoplastic angiogenesis. VEGF overexpression and elevated serum levels of this cytokine have been observed in several malignancies [2].

The *VEGF* gene is located on chromosome 6 (6p12.1) and is highly polymorphic. The −1154 G/A, −460 T/C, and +405 G/C *VEGF* polymorphisms have been reported as functionally relevant and associated with an increased risk of development of various inflammatory or neoplastic processes [16, 26].

The pathogenesis of basal cell carcinoma (BCC)—the most common malignancy in Caucasian populations is complex, but is strongly associated with environmental and genetic factors. Although the genetic background of BCC has been analyzed in some research, the mechanism of BCC pathogenesis is not yet fully understood. To the best of our knowledge, *VEGF* gene polymorphisms have not been explored to date in this context [12].

In this study, three polymorphisms in the *VEGF* gene (−1154 G/A, −460 T/C, and +405 G/C) were assessed in relation to the risk of BCC incidence in a population from northern Poland and some clinical aspects of the malignancy. In addition, VEGF serum levels of patients with BCC were compared with those of a control population.

## Materials and methods

### Patients and controls

The study included 180 unrelated patients with BCC and of mean age 68.9 ± 11.8 (96 women, 84 men) and 215 healthy, unrelated age- and sex-matched volunteers (Table 1). None of the subjects were organ transplant recipients, none were being treated with immunosuppressive drugs, and none suffered from any systemic inflammatory disease or malignancy. All subjects were exclusively of Eastern European/Polish descent.

**Table 1:** Characteristics of the BCC patients investigated

	Males	Females	Overall group
	84 (46.7 %)	96 (53.3 %)	180
Age (mean ± SD)	69.6 ± 9.9	68.5 ± 13.1	68.9 ± 11.8
<60 years	13 (37.1 %)	22 (62.9 %)	35 (22.6 %)
>60 years	59 (49.2 %)	61 (50.8 %)	120 (77.4 %)
Tumor size (cm)			
≤1	27 (35.1 %)	50 (64.9 %)	77 (48.4 %)
>1	44 (53.7 %)	38 (46.3 %)	82 (51.6 %)
Recognition:			
BCC	71 (45.8 %)	84 (54.2 %)	155 (90.6 %)
BCC recurrence	7 (43.7 %)	9 (56.2 %)	16 (9.4 %)
Number of tumors:			
One tumor	62 (43.1 %)	82 (56.9 %)	144 (81.8 %)
Multiple tumors	19 (59.4 %)	13 (40.6 %)	32 (18.2 %)
Location:			
Area exposed to UV	58 (42.6 %)	78 (57.4 %)	136 (85.0 %)
Area not exposed to UV	14 (58.3 %)	10 (41.7 %)	24 (15.0 %)

The study was approved by the local research ethics committee of the Medical University of Gdańsk.

Patients with BCC were subclassified by tumor site, tumor size, age, and recurrence.

### VEGF genotyping

The *VEGF* polymorphisms at positions −1154 and +405 were analyzed using the amplification refractory mutation system polymerase chain reaction method (ARMS-PCR), as has been described in [7].

To assess *VEGF* gene polymorphisms at position −460, we employed the polymerase chain reaction restriction fragment length polymorphism method (PCR–RFLP) according to the method described by Kuo et al. [11].

### VEGF serum level analysis

Serum concentrations of VEGF were measured in 135 patients with BCC and in 62 unaffected subjects. The median values for the protein concentration were not affected by the age or sex at enrollment in either the BCC cases or the controls.

Serum levels of VEGF protein were measured using the ELISA test (The Quantikine Human VEGF Immunoassay, R&D Systems, Inc., Minneapolis, USA), following the manufacturer’s instructions.

### Statistical analysis

The *χ* ^2^ analysis was used to compare the observed number of genotypes with that expected for a population in a Hardy–Weinberg equilibrium. The *χ* ^2^ analysis was also employed to test the significance of the differences in the observed alleles and genotypes between groups. A logistic regression model was used to calculate the odds ratios (ORs) and the 95 % confidence intervals (CIs). The Mann–Whitney *U* test was used to compare the mean values, and the correlation was determined using mean Spearman coefficient values. Analyses were performed using the Statistica 8.0 software package (StatSoft, Inc., 2008). *p* < 0.05 was considered statistically significant. Haplotype estimation was carried out using the Phase v2.1 software package.

## Results

### Analysis of VEGF polymorphism

The distribution of the *VEGF* genotypes was consistent with a Hardy–Weinberg equilibrium only in the control group.

The *VEGF* genotype frequency of each of the groups is shown in Table 2.

**Table 2:** Genotypes and alleles frequencies for VEGF −1154 G/A, −460 T/C and +405 G/C in patients with BCC and control subjects

Genotypes and alleles	Controls	BCC	OR (95 % CI), *p*	aOR (95 % CI), *p*
−1154	*n* = 215	*n* = 180		
GG	75 (34.9 %)	64 (35.6 %)	NS	NS
GA	103 (47.9 %)	111 (61.7 %)	**1.75 **(1.27–2.89), *p* = **0.006**	**1.87** (1.75–1.99), *p* <** 0.00001**
AA	37 (17.2 %)	5 (2.8 %)	**0.14 **(0.04–0.32), *p* < **0.0001**	**0.09** (0.08–0.11), *p* <** 0.00001**
GG + GA vs AA	178 (82.8 %) vs. 37 (17.2 %)	175 (97.2 %) vs. 5 (2.8 %)	**7.28** (3.07–25.40), *p* < **0.0001**	**10.52** (9.02–12.27), *p* <** 0.00001**
	*n* = 430	*n* = 360		
G	253 (58.8 %)	239 (66.4 %)	*p* = 0.03*	*p* = 0.03*
A	177 (41.2 %)	121 (33.6 %)		
−460	*n* = 215	*n* = 180		
TT	59 (27.4 %)	42 (23.3 %)	NS	NS
TC	119 (55.3 %)	110 (61.1 %)	NS	NS
CC	37 (17.21 %)	28(15.6 %)	NS	NS
	*n* = 430	*n* = 360		
T	237 (55.1 %)	194 (53.9 %)	NS	NS
C	193 (44.9 %)	166 (46.1 %)	NS	NS
+405	*n* = 215	*n* = 180		
GG	123 (57.2 %)	90 (50.0 %)	NS	NS
GC	86 (40.0 %)	85 (47.2 %)	NS	NS
CC	6 (2.8 %)	5 (2.8 %)	NS	NS
	*n* = 430	*n* = 360		
G	332 (77.2 %)	265 (73.6 %)	NS	NS
C	98 (22.8 %)	95 (26.4 %)	NS	NS

Bold values are statistically significant (*p* < 0.05)

*BCC* basal cell carcinoma, *OR* crude odds ratio, *aOR* adjusted odds ratio, *CI* confidence interval, *NS* not significant

* *χ* ^2^ Pearsona

Allele frequencies did not significantly differ across all BCC patients and controls at the −460 and +405 loci, but at position −1154, the G allele was observed statistically more frequently among patients (*p* = 0.03).

The presence of the G allele (GA or GG) in −1154 *VEGF* polymorphism was associated with an increased risk of developing BCC (OR = 7.28, *p* < 0.0001). Furthermore, the carriers of the AA genotype in −1154 *VEGF* polymorphism showed a significantly reduced risk of BCC (OR = 0.14, *p* < 0.0001).

Haplotype frequencies for the −1154 G/A, −460 T/C, and +405 G/C polymorphism of *VEGF* are shown in Table 3. These data demonstrate that the haplotype frequencies differ significantly between the BCC cases and the controls (*p* = 0.03).

**Table 3:** Haplotype frequencies for VEGF −1154 G/A, −460 T/C and +405 G/C estimated using PHASE version 2.1

Haplotype −1154, −460, +405	Controls (*n* = 430)	BCC (*n* = 360)	OR (0.95 % CI), *p*	aOR (95 %CI), *p*
ACG	114 (26.5 %)	101 (28.1 %)	NS	NS
GCG	71 (16.5 %)	62 (17.2 %)	NS	NS
GTG	108 (25.1 %)	97 (26.9 %)	NS	NS
ATG	39 (9.1 %)	6 (1.7 %)	**0.17** (0.07–0.40), *p* = **0.00001**	**0.21** (0.18–0.23), *p* <** 0.00001**
GTC	72 (16.7 %)	80 (22.2 %)	**1.69** (1.09–2.47), *p* =** 0.013**	**1.88** (1.76–2.02), *p* < **0.00001**
ATC	18 (4.2 %)	11 (3.1 %)	NS	NS
ACC	6 (1.4 %)	3 (0.8 %)	NS	NS
GCC	2 (0.5 %)	0 (0.0 %)	NS	NS

Bold values are statistically significant (*p* < 0.05)

It was shown that the GTC haplotype of *VEGF* predisposes to BCC development (OR = 1.69, *p* = 0.013), while the presence of the ATG haplotype significantly reduces that risk (OR = 0.17, *p* = 0.00001).

The higher frequency of the GG genotype in −1154 *VEGF* polymorphisms was observed in patients with tumors localized on unexposed areas, as compared to those with tumors on exposed areas (45.8 vs. 33.1 %). The A allele was observed more frequently in the case of BCC on sun-exposed skin (66.9 vs. 54.2 %).

Age analysis of the BCC patients shows a significantly higher occurrence of the GG genotype in patients older than 60 (40.8 vs. 11.4 %; *p* = 0.001).

### VEGF serum level

**Fig. 1:**
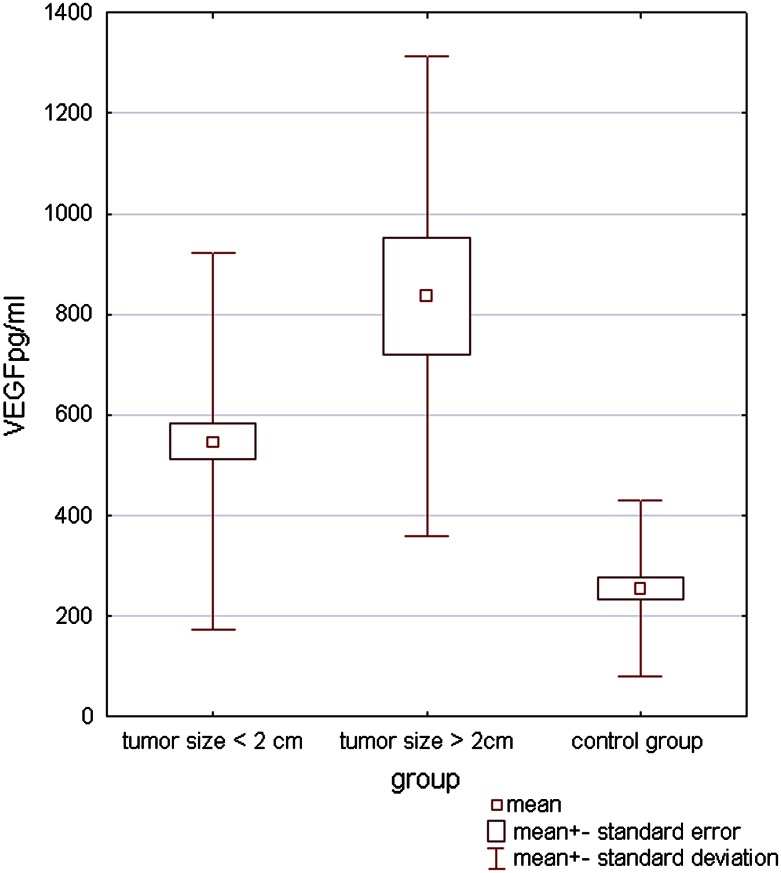
Correlation of VEGF serum levels with tumor size (cm)

We found significantly increased VEGF serum levels among BCC patients, in comparison with healthy controls (mean 596.7 ± 393.5 pg/ml; range 60.1–931.4 vs. 255.9 ± 174.6 pg/ml; range 42.2–553.0 pg/ml; *p* < 0.0004). Moreover, higher levels of VEGF were observed in patients with tumors localized on unexposed areas (764.49 ± 365.18 vs. 559.98 ± 397.66; *p* = 0.023) and with size greater than 2 cm (836.1 ± 476.9 vs. 547.4 ± 373.8; *p* = 0.0052). Serum levels of VEGF significantly correlated with tumor size: *r* = 0.41, *p* < 0.0001 (Fig. 1).

There was no significant association between patients’ ages and their VEGF serum levels. No correlations were demonstrated between the analyzed *VEGF* polymorphisms and the VEGF serum levels (*p* > 0.05 for all comparisons).

## Discussion

Ultraviolet radiation plays a crucial role in the pathogenesis of skin cancers. However, the significant number of tumors that arise on areas of the skin that are not exposed to the sun suggests that other factors also play a role in the pathogenesis of BCC. An association between BCC development and personal and family history of skin cancer has been demonstrated [13].

Genetic studies in patients with BCC tend to focus on the genes involved in DNA repair, defense against oxidative stress and other mutagens, immune modulation, tanning, and other biochemical activities. To date, these studies have provided inconsistent results, perhaps indicating variability in the selection and numbers of patients and controls, or reflecting different effects in different cancers [10, 12, 22, 24].

Many potent angiogenic factors have been described in malignancies. Recent data strongly indicate that VEGF is the main protein involved in tumor growth and metastasis. Previous studies performed on a number of types of tumors (breast, oral, lung, colorectal, and prostate cancer) have demonstrated that this cytokine is overexpressed and that its enhanced level correlates with poor prognosis [3, 9, 20, 23, 26].

In many different types of malignancies, the *VEGF* gene single nucleotide polymorphisms were associated with disease susceptibility, disease progression, and resistance to treatment [1, 6, 8, 11, 14–16, 25]. The role of VEGF and its gene polymorphisms in non-melanoma skin cancers has not been studied so far.

This study has evaluated the potential impact of three *VEGF* gene polymorphisms on the presence of BCC and on some of its clinical aspects. We have found only an association between −1154 G/A *VEGF* polymorphism and BCC development. The presence of the G allele correlated with an eightfold higher risk of malignancy. The AA genotype was associated with a reduced risk of BCC. A similar observation was made by McCarron et al. [17], who demonstrated that the AA genotype in this polymorphism reduces the risk of prostate cancer (OR, 0.45). Li et al. [14] confirmed this phenomenon in an ovarian cancer cohort study. Our results suggest a potential protective effect of the AA genotype in the −1154 G/A *VEGF* polymorphism against BCC development. This finding is in accordance with the results of Howell et al. [6], who showed that the AA genotype is associated with a thinner primary vertical growth phase of cutaneous melanoma, in contrast to GG genotype carriers, who showed thicker tumors. Based on these data, it may be concluded that the presence of the AA genotype may be correlated with slower disease progression, probably thanks to its influence on the encoding of protein expression. However, many environmental factors may affect the regulation of gene expression.

We have also shown a higher frequency of the GG genotype in −1154 G/A *VEGF* polymorphism in BCC cases that are localized on an unexposed area. This interesting observation might indicate, apart from UVR, the role of VEGF polymorphism in BCC pathogenesis.

The fact that GG genotype occurs more frequently in patients older than 60 is probably coincidental, and indicates that further investigation is required on a larger population.

In various cancers, a positive correlation was revealed between VEGF serum levels and tumor size, as well as between lymph node involvement and the presence of metastases [26].

We have also found higher VEGF serum levels in BCC patients, compared with the controls. Moreover, a positive association between VEGF serum levels and tumor size and site was found which may underline the role of VEGF in tumor aggressiveness. In some reports, the impact of various *VEGF* polymorphisms on cytokine synthesis was shown [5, 20]. Although we have not found the analyzed polymorphisms to have a significant influence on cytokine serum concentrations in patients, we have demonstrated this relationship in controls, showing that the presence of the GG genotype −1154 in G/A VEGF polymorphisms is linked to higher VEGF serum concentrations. These results are consistent with some other reports [21, 29].

Recently, novel therapeutic strategies including anti-VEGF agents (bevacizumab, aflibercept, regorafenib, and ramucirumab) have been introduced for metastatic renal cell carcinoma and for nonsquamous non-small-cell lung cancer, melanoma, glioblastoma, pancreatic cancer, and metastatic colorectal cancer [7, 19]. Our results support the thesis that VEGF may be a good indicator of disease severity and localization, and it is likely that topical therapies directed against VEGF or its receptors will constitute new approaches to BCC treatment.

Some studies have shown that ACE inhibitors can significantly inhibit tumor growth and angiogenesis in some malignancies, and suppress VEGF [27, 28]. Napoleone et al. [18] demonstrated that downregulation of rennin–angiotensin system by ACE inhibitors and angiotensin receptor blockers inhibits tissue factor and VEGF expression in highly metastatic breast cancer cells.

Yapijakis et al. [27] analyzed a functional polymorphism in the ACE gene, which affects its transcription, with risk for BCC. They showed that ACE polymorphism is associated with decreased risk for BCC in ID heterozygotes. That novel observation suggests that rennin–angiotensin system inhibition may be considered also as a new potential strategy for combined modalities in BCC treatment.

In conclusion, our results testify to the importance of −1154 G/A *VEGF* gene polymorphisms in altering the risk of BCC within a population from northern Poland. The interactions that determine other VEGF polymorphisms, skin VEGF expression in tumors, solar UV exposure, and various environmental factors should be performed in future studies, to confirm the links between gene polymorphisms and BCC risk.
